# Exploring the Ethical and Practical Considerations of Artificial Intelligence in Real-World Health Care Settings: Stakeholder Focus Group Study

**DOI:** 10.2196/85163

**Published:** 2026-04-02

**Authors:** Carmen Wendy Ulizio, Devika Dua, Naya Meenkashi Mukul, Santosh Areti, Kristin Kostick-Quenet, Vasiliki Nataly Rahimzadeh

**Affiliations:** 1 Baker Institute for Public Policy Rice University Houston, TX United States; 2 Center for Medical Ethics and Health Policy Baylor College of Medicine Houston, TX United States

**Keywords:** AI ethics, AI guidance, AI implementations, AI, artificial intelligence, focus group, qualitative study

## Abstract

**Background:**

Artificial intelligence (AI) technologies continue to transform how we research human disease, diagnose and treat patients, and operate hospitals. However, emerging ethical dilemmas surrounding their design, use, and oversight demand both policy attention and empirical research.

**Objective:**

This study aims to explore current AI development, integration, and use activities across the Texas Medical Center (TMC), the largest medical center in the world, and identify emerging ethical priorities.

**Methods:**

We conducted a total of 3 qualitative focus groups via Zoom (Zoom Video Communications, Inc) between May and June 2025 to gauge the perspectives of 19 clinicians, developers, administrators, and patient advocates on core aspects of clinical AI tools at the point of care.

**Results:**

Participants described current development and deployment of AI tools across the TMC, with areas of high potential focused on extending clinical expertise, reducing administrative burden, and improving cross-specialty collaboration. However, they also identified many challenges, including significant barriers to accessing quality datasets for training, insufficient systematic governance on the validation, auditing, and use of AI tools in the clinic, and limited patient involvement in AI development decisions. Discussion on validation of models occurring primarily in well-resourced locations like the TMC raised worries about a potential digital divide in health care. These concerns were heightened for practitioners working in safety-net hospitals and in other underresourced health care settings. Participants also highlighted that discussions on AI ethics at the development stage are currently lacking and suggested embedding bioethicists into development teams to account for this issue. Clinicians and patient advocates differed in their views on patient notification about the use of AI at the point of care, justifying future research on this question. Accountability also remained an unresolved issue, with participants split on whether the provider should take full responsibility for any patient care errors resulting from AI.

**Conclusions:**

These contributions identify the ethical tensions currently occurring in the real-world daily lives of professionals involved with health AI within a large regional academic medical center. Addressing these challenges will require AI-specific governance that ensures contextual validation, easy access to data, independent auditing, meaningful stakeholder involvement, and support and education for frontline users who must integrate these tools into their daily practice.

## Introduction

Advances in artificial intelligence (AI) and machine learning (ML) have touched nearly every sector, from industry and transportation to communication and health care. Within health care specifically, AI has grown significantly from basic image analysis to more complex applications [1]. Currently, AI and ML technologies have been shown to be more accurate at detecting cancerous lesions than trained radiologist residents [2,3]. Furthermore, implementing AI to help streamline diagnostic testing and monitor service use saves hospitals millions of dollars annually [4,5]. AI’s powerful analytic capabilities have also helped scientists study new genes associated with disease and understand how environmental and behavioral factors influence disease risk and progression [6], with more work still to be done [7].

Despite these scientific developments and operational gains, questions remain about how AI can be integrated into clinical settings safely [8-11], ethically [12-16], and equitably [17-20]. Previous work addressing these questions has raised many ethical concerns, including fairness, privacy, trustworthiness, accountability, explainability, validation, and more [21]. These concerns about health AI stem in part from the enormously rich, yet sensitive nature of health data needed to effectively train AI models. AI systems are data-hungry and can only be as safe and effective as the data upon which they are trained [22]. Lack of quality and representative datasets can lead to biased AI tools that threaten patient safety and compromise patient trust [23]. Previous work has also identified other data-independent factors that can impact patient safety, including overreliance, inadequate clinical integration, lack of standardized deployment guidelines, and insufficient monitoring [24]. There is also ongoing ethical debate about whether and how to consent patients about the use of AI tools in clinical care [25] and what impacts health AI tools have on the patient–provider relationship. Current research also demonstrates how recurring issues like lack of transparency, insufficient governance, and minimal patient involvement are undermining patient trust in AI tools [26]. These issues are deeply rooted in the values and ethical principles that ground our health care systems; therefore, addressing these concerns requires not only better technology but also humanities-based research and policy attention. However, there are significant knowledge gaps about how to best center the values of patients as well as providers in ethical AI development, implementation, and evaluation in clinical care [27].

Such questions are becoming increasingly apparent and more complex within the Texas Medical Center (TMC), the world’s largest medical complex, and a nationally recognized hub for clinical innovation. The TMC is home to more than 60 member institutions, including Rice University, Baylor College of Medicine, MD Anderson Cancer Center, Houston Methodist Hospital, and Texas Children’s Hospital, all of which are actively involved in the development, testing, or implementation of AI and ML technologies [28]. Despite these activities, opportunities for cross-institutional learning, collaboration, and engagement around the development, implementation, and ethical evaluation of AI technologies have been severely limited across the TMC. Texas’s distinct regulatory environment is also increasingly shaping how stakeholders within the TMC adopt and use AI tools in health care. The state’s recently established Texas Responsible Artificial Intelligence Governance Act aims to support the safe development and validation of AI systems for real-world clinical settings while facilitating partnerships that drive AI innovation [29]. Additionally, the new Texas Senate Bill 1188 now requires health care workers to disclose any use of AI for diagnostic purposes and closely review all AI-generated records, establishing expectations for how clinicians must use AI tools [30]. Together, these policies create a unique regulatory landscape that encourages rapid development and innovation while simultaneously establishing rules that promote consistent oversight of AI use in medical decision-making.

Prior studies have also investigated how safety-net and rural providers in Texas perceive health AI and highlighted concerns such as trust, institutional readiness, and inequity [31]. However, research into the perspectives of stakeholders actively working with AI in large TMCs is lacking. Understanding the viewpoints of those actively developing and making decisions about AI use at the point of care is vital to gaining a more comprehensive picture of AI’s day-to-day impact in clinical settings. Using a qualitative approach, this study seeks to address this knowledge gap and situate a global conversation on health AI within a regional academic medical center and among stakeholders directly engaging with the development, application, and ethical implications of AI in health care. While much of the existing health AI ethics literature focuses on the isolated perspectives of clinicians or developers, this project convened a diverse group of participants, including AI developers, health care providers, institutional administrators, computational scientists, bioethicists, and patient advocates currently working within the TMC to participate in a series of moderated virtual focus groups. The study was guided by three primary objectives (1) to identify the types of health problems and clinical challenges that AI tools are currently being developed to address across the TMC; (2) to examine how these tools are currently being implemented, experienced, and used in research and care delivery, as well as the specific concerns that arise from this use; and (3) to analyze the broader ethical dilemmas associated with AI integration in health care and propose policy recommendations and strategies to address these issues. Importantly, as many of the stakeholders interviewed are individuals who must ultimately execute AI policies, they are well-equipped to identify potential problems and areas for improvement within clinical workflows. By engaging a broad range of stakeholders, we aim to offer a map of promising use cases, identify implementation challenges, and formulate institutional strategies and ethical recommendations that are grounded in real-world experience and practice-based realities within a major medical center in Texas, where state legislation on AI is among the nation’s most advanced.

## Methods

### Study Design

The research team conducted qualitative focus groups to glean a deeper understanding of health AI development, use, and integration across the TMC and develop ethical local guidance. These focus groups aimed to advance research in health AI ethics by fostering discussions across developer, user, and patient communities. In so doing, the study aimed to elicit diverse insights at each stage of the AI lifecycle, from conceptualization to development and deployment.

- Focus group 1: development (what problems are researchers, developers, and health care professionals looking to AI tools to solve?)
- Focus group 2: use (how are researchers, developers, and health care professionals across the TMC using health AI?)
- Focus group 3: integration (what ethical issues are researchers, developers, and professionals encountering when applying health AI in the real-world?)

### Participant Recruitment and Participation

Eligible participants actively worked within the TMC. Lists of potential participants were developed using websites of universities and hospitals within the TMC, as well as lists of speakers at local AI in health care conferences and symposia. Purposive sampling helped the study team identify eligible participants with prior leadership experience in health AI. To include the perspectives of patient communities, individuals from patient advocacy groups with branches in Houston, Texas, were selected. Each selected participant was emailed once a week for 4 weeks until they filled out an availability questionnaire or requested to be removed from the study (Table 1). Participant roles were assigned based on the participant’s self-identified primary role, although some participants occupied multiple stakeholder roles.

**Table 1:** Framework of participant recruitment and attendance for each focus group.

Characteristic	Focus group 1	Focus group 2	Focus group 3
Aim	Development	Use	Integration
Participants contacted, n	79	53	41
Participants confirming attendance, n	8	7	10
Participants on call, n	8	3	8
Participation rate, n (%)	8 (10.1)	3 (5.6)	8 (19.5)

### Data Collection and Analysis

All data collection was done via focus groups that were conducted virtually on Zoom (Zoom Video Communications, Inc) and lasted 90 minutes each. Moderators facilitated introductions and guided participants through a series of structured questions related to health AI development, use, or integration. Facilitators followed up wherever necessary (ie, clarification, gleaning further information, etc). Questions focused on participant expectations, experiences, perceptions, and suggestions across the development, use, and integration of health AI across the TMC. The complete focus group question guides are available in Multimedia Appendices 1-3. Following each focus group session, recordings were transcribed using Microsoft Copilot’s AI-assisted transcription service and then cleaned by a member of the research team to ensure accuracy. Any identifiable information was removed from the transcripts prior to analysis. Additional participant quotations from the focus groups are available in Multimedia Appendix 4.

**Figure 1:**
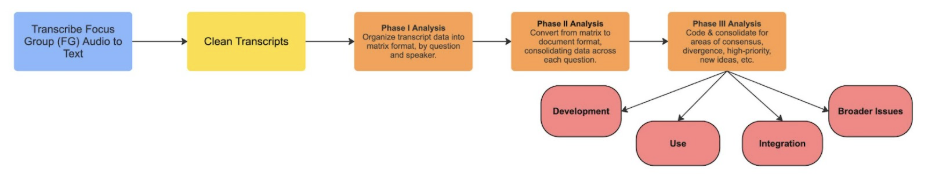
Workflow of data collection, preparation, and analysis.

The research team used a rapid qualitative analysis approach conducted in 3 phases to analyze transcript data and extract key themes (Figure 1). First, transcripts were converted into matrices organized by question asked and stakeholder role, with transcript text synthesized into concise, reflective summaries that preserved the speaker’s meaning and maintained original quotes to illustrate key points (Phase I). These matrices were then transformed into thematic documents that summarized responses by focus group question, prioritized high-impact quotes, and presented findings in a transferable format (eg, bullet form; Phase II). Finally, the Phase II summaries were organized to reveal areas of consensus, divergence, and new ideas, as well as high-priority concerns, recommendations, lightly discussed topics, and commonly acknowledged issues. Key themes were then extracted and written up in paragraph form (Phase III). Any loosely defined points or unresolved issues were deliberated in research team meetings and resolved by consensus. Deductive coding was applied throughout, using the structured focus group guide as a preliminary codebook and aligning findings with known themes from the health AI ethics literature and prior AI roundtable discussions across the TMC, including fairness, explainability, bias, accountability, transparency, and others [32]. Each focus group was reviewed by at least 2 analysts, and all members of the research team contributed to the synthesis of the dataset. This approach allowed the research team to harness stakeholder perspectives on the current key policy issues facing the TMC while simultaneously allowing new data themes to emerge.

### Ethical Considerations

This study was approved by the Rice University Institutional Review Board (protocol number IRB-FY2025-148). Prior to each session, participants (Table 2) reviewed and signed a consent form detailing the focus group’s purpose, procedures, risks, and benefits and prior to proceeding with the focus group on Zoom. Participation in the focus groups was voluntary, and participants were informed that they could withdraw from the study at any time. Following the focus group all data was deidentified. To encourage participation, a small honorarium of US $75 per participant was offered following the end of the focus group.

**Table 2:** Participant roles within each focus group.

Focus group	Providers, n	Developers, n	Ethicists, n	Administrators, n	Data curators, n	Patient advocates, n	Policy experts, n
1	1	5	—^a^	1	1	—	—
2	2	1	—	—	—	—	—
3	2	2	1	—	—	2	1

^a^Not applicable.

## Results

### Overview

A deductive analysis of participant responses revealed recurring themes as well as unique insights that reflect the diversity of expertise and experience among participants. These findings are organized by (1) AI applications, (2) implementation experiences, and (3) ethical challenges and recommendations. Within each section, we highlight key themes, AI tools discussed (Table 3), as well as areas of convergence and divergence across stakeholder perspectives.

**Table 3:** AI tools mentioned in stakeholder discussions and their transferable insights.

Tool mentioned	Section	Theme	Insights
CAP^a^ Tool	AI^b^ applications	Extend clinical expertise	Highlights how the greatest areas of AI potential lie in extending niche expertise to areas where that expertise would not otherwise exist.
Frailty Assessment Tool	AI applications	Enable cross-specialty collaboration	Demonstrates AI’s ability to mediate disputes between specialties using evidence-based practice. Supports the whole patient’s health and evaluation as opposed to specialty-specific goals.
Best Care Choices for My Patient	AI applications	Synthesize large datasets	Shows how AI can use large, real-world datasets to answer “edge case” clinical questions, especially in time-sensitive situations.
EHR^c^ LLMs^d^	AI applications	Synthesize patient information	Emphasizes physician’s need for AI to summarize patient histories, trend laboratory values, and overall improve the usability of EHRs.
Patient Portal Summarizers	AI applications	Simplify out-of-office communication	Demonstrates potential role of AI in helping patients understand medical language, laboratory results, and care instructions, while easing provider workload by reducing message volume.
Continuous Care Patient Assistant	AI applications	Supporting patient empowerment and care beyond the clinic	Highlights how AI can be used to provide patient care outside of the doctor’s office and answer previsit and postvisit clarifying questions. These types of tools will help patients ask more informed questions, reduce uncertainty of postvisit instructions, and extend care in a 24/7 manner without increasing physician burden. However, concern that current AI tools like ChatGPT are not fully equipped for this demonstrates a need for AI tools specifically designed for this purpose and with the patient in mind.
OpenEvidence	Implementation experiences	Decision-making support	Illustrates how AI decision-making support tools are currently used as a nonjudgmental colleague to quickly synthesize evidence in clinical practice. Its use also demonstrates the need for providers to build their own trust with support tools before implementing them into their practice and for trainees to learn how to prompt and evaluate AI tools against their own clinical reasoning rather than just growing reliant on the tool’s output.
EPIC Sepsis Detection Model	Implementation experiences	AI deployment failures	Shows how poor validation across more diverse settings can lead to alarm fatigue and loss of trust not just in one AI tool but in any future tools, thus reducing overall effectiveness. This illustrates the need for governance that oversees context-specific validation and continuous monitoring to prevent these outcomes.

^a^CAP: Child Abuse Protection.

^b^AI: artificial intelligence.

^c^EHR: electronic health record.

^d^LLM: large language model.

### Section 1: AI Applications

A primary focus of discussion, particularly among developers, was priority areas for AI innovation and ideas for AI applications. Experts generally agreed that the most valuable AI tools would be those that extend clinical expertise to underserved areas. One developer gave an example where their hospital developed an initiative to deliver AI-enabled Child Abuse Protection team services to physicians in areas where Child Abuse Protection professionals did not exist or were severely limited. They noted that:

The greatest developments that we can work on are the ones where we can encapsulate our expertise and push them out to areas where that expertise doesn't exist.

Developer, focus group 1

Other high-priority AI applications discussed included tools that enable cross-specialty collaboration and mediated interspecialty disagreements. For instance, another developer described the creation of an AI-driven frailty assessment tool designed to assist non-geriatricians, such as anesthesiologists and surgeons, in determining surgical fitness. This tool aims to optimize overall patient health as opposed to specialty-specific goals such as cardiologists’ focus on heart health.

Similarly, experts highlighted AI applications in complex medication management, where disagreements often arise between specialists prioritizing different aspects of patient care. In these cases, AI systems aggregate and present relevant clinical evidence, such as drug interaction risks and comparative outcome data, in a standardized format. Currently, Epic, a widely used electronic health record (EHR) system across the TMC, has also launched a Best Care Choices for My Patient initiative that uses its Cosmos research database to answer “edge,” pressing questions in active patient care, such as, “If I use antihypertensive A, what’s the rate of stroke in the following five years versus using antihypertensive B?” By offering an objective synthesis of information, these tools help care teams move beyond specialty-specific disagreements and time-sensitive decisions, facilitating more balanced patient-centered decisions. While specific tools may vary across specialties and clinical settings, these examples collectively demonstrate how AI can support multidisciplinary decision-making by synthesizing expertise across areas of clinical expertise.

Looking to the future, experts highlighted AI’s potential to improve health care workflows by optimizing efficiency, reducing costs, and alleviating administrative burdens. One developer noted that AI’s greatest potential is anywhere that “efficiency can be improved, and the regulatory obstacles can be surmounted” (Developer, focus group 1). Tasks discussed that fell into this category included automating time-intensive tasks such as predicting patient no-shows by demographic factors, automating summaries of patient charts, sending referral letters to other providers, predicting outcomes using past test results to reduce the need for and cost of new test orders, and using ambient-listening to create visit notes. These examples were offered as opportunities to improve physician productivity while lowering workflow costs.

Another top use case centered on improving access to information within EHRs. Participants noted that physicians’ current expectations of the EHR are alarmingly low. In response, physician participants expressed the potential value of integrating large language models (LLMs) to reduce documentation burdens and enable easier synthesis of patient information. For example, 1 physician participant expressed:

What I find the most frustrating thing about working in the EMR is that there is so much information there, and despite [that]... you still can't always consistently access it…If it takes AI for us to be able to do that or to be able to search even slightly more complex things…it really would make a huge difference.

Provider, focus group 2

Many physicians also believe that AI can be harnessed to maintain quality patient care outside of the clinic and guide patient consultations. Some suggested promising examples of tools in this domain include those that improve telehealth efficiency and patient-portal communication. Specifically, tools that could assist providers in generating responses to patient questions and messages or summarize patient-portal communications for easier physician response. One participant also described the growing popularity and potential of tools that improve both previsit and postvisit interactions. For example, a useful tool could allow a patient to ask previsit questions about symptoms they may be experiencing or research treatment options for, situationally prompting necessary visits. Then, after their visit, patients could ask the AI practical questions they did not think of when they were with their provider. One physician added:

Questions come up not only at the time that you're meeting with the doctor... You talked to your family, or you had a chance to think about it, and then you're like, wait a second. You know how many pounds can I lift after this surgery? I forgot what the doctor said.

Provider, focus group 2

Such questions could be addressed by AI tools postvisit to reduce provider workloads and give patients easy access to medical information, thus improving the quality of patient care outside the physical clinical setting. Physicians who participated in our focus group also shared how they hoped AI could assist in health monitoring activities outside of the doctor’s office, such as taking biometrics, performing screenings, or providing patients with relevant health information so they can receive care year-round, not just when they visit the doctor. While harnessing AI outside the clinical setting may have many advantages—including prompts that motivate patients to seek timely care and give answers postvisit questions—participants noted important limitations. Participants specifically raised concerns about how generative AI tools, such as ChatGPT (OpenAI), can overly generalize and lack clinical accuracy. Therefore, the role of these tools in disseminating information to and interacting with patients was considered risky. To account for these issues, 1 developer noted that, “we need to think about converting from the model to a product that the patient can use” (Developer, focus group 3), underscoring the need for new AI tools designed specifically with patients in mind.

Together, these AI applications reflect an overall optimism regarding AI’s ability to extend clinical expertise, synthesize important information in clinical care, and support patient care beyond physical health care settings. However, as the subsequent section demonstrates, real-world stakeholder experiences with the implementation of these tools reveal practical and ethical tensions that emerge once tools are deployed in real-world settings.

### Section 2: Implementation Experiences

Discussion around how current AI tools are being implemented, evaluated, and experienced at the point of care was prominent within the focus groups. More specifically, participants highlighted the ethical and practical concerns that are arising in this use. A large portion of this discussion on current use involved the utility of OpenEvidence, a clinical decision-support tool that leverages AI to synthesize the latest medical research and make individual care plans. These experts described using OpenEvidence in the same way they would consult an informed colleague, using it to support differential diagnoses and facilitate conversation-based decision-making using specific parameters like laboratory values. Participants considered the tool trustworthy because it provides citations for information and does not hallucinate like other generative AI tools, including ChatGPT. Participants also emphasized its value in efficiently synthesizing medical literature and providing a faster and more targeted alternative to searching PubMed or Google that better contextualizes the patient’s presentation in ways that traditional search engines cannot.

However, while participants generally agreed that AI can augment clinical decision-making, they also stressed that nothing could replace authentic clinical experience and judgment of real people. Considering the alarming ease with which AI can be integrated as a replacement for clinical decision-making, many physicians noted the challenge of balancing their own clinical judgement with the potential of AI tools. Some physicians raised the concern of immediately trusting AI without validating it for themselves and highlighted the need for physicians to develop personal trust with an AI tool before using it. For example, 1 physician described how they tested OpenEvidence themselves, stating:

So, asking it questions in the way that I was framing them and seeing if it was coming up with similar responses or if it was coming up with new ideas...after that, I started to kind of feel like, OK. Here's the type of question that I feel like I can trust it with based on the relationship…But I think that there is a real danger and just kind of jumping in immediately, trusting it, not having any kind of sense of how to craft prompts or where the information is coming from.

Provider, focus group 2

In addition to developing personal trust with AI tools, many clinicians across our focus groups also emphasized the importance of retaining decision-making authority in the care of their patients, especially because the outputs of predictive AI models do not always give a black-and-white answer. Instead, most of the time, it will give the user a risk score (ie, “you have a —% risk of having a bad outcome after this surgery based on your frailty score”). Experts highlighted that thresholds for action or intervention according to these values or risk scores, need to be well defined before a physician can make a decision for their specific patient. One physician shared:

So, the model just tells you the likelihood...of something happening. Then we have to define what those thresholds are. And I think what is very important is what are we going to do about it if it meets a certain threshold? What is the action plan and how much are we willing to be wrong in those.

Provider, focus group 2

However, participants noted that the black-box nature of many AI tools makes it difficult for physicians to verify informational outputs and determine thresholds for action, further calling for transparency and explainability of AI systems.

Another persistent view among participants was that, despite the select examples described previously, overall, AI currently lacks impact in actual clinical practice. Some experts stated that this is because advancing beyond Phase I, or AI model development, is exceptionally challenging, with 1 developer noting that:

[The] majority of the AI models nowadays [are] stopped, ended at phase one. People published [a] paper [and] call it from there. It's extremely challenging to translate the AI model to phase two.

Developer, focus group 3

Even those technologies that do make it to Phase II, deployment in clinical settings, have limitations. For example, experts discussed how some models that have been rolled out, including LLMs for mental health consultations, are done so without proper validation and testing, which leads to problematic long-term outcomes. Another example physicians mentioned was the underwhelming impact of a sepsis detection model by the EHR manufacturer Epic, which did not perform well when applied to diverse patient groups. These results are causing hesitancy among providers at the hospital in deploying other AI models offered by Epic, further demonstrating how lack of contextual validation can harm physician trust in AI systems. Moreover, physicians highlighted their concerns for alarm fatigue with the sepsis alerts, with 1 physician stating:

Everybody hates the sepsis alerts. It's just, you know, you've figured out what you need to click to get past it....I think the sepsis alert in EPIC and [Health System] has lost everyone's trust. So, I think that's a danger of other AI tools as well.

Provider, focus group 2

Experts noted that this model brought awareness to the need for thorough validation and representative data for AI models as well as to potential future problems of AI model deployment.

Across these implementations and use experiences, participants repeatedly spoke on concerns regarding trust, limited deployment success, and validation. The recurrence of these themes underscores that these challenges are not just isolated incidents but broader structural issues in clinical deployment that bring up significant ethical issues, as discussed below.

### Section 3: Ethical Challenges and Recommendations

Discussion on the development and integration of AI systems not only raised technical challenges but also broad ethical concerns among focus group participants. Central themes included how AI models are contextualized in practice, the barriers posed by limited access to data, and the challenges of maintaining auditability over time. Experts also discussed the importance of keeping ethics and patient-centered care in mind throughout the AI lifecycle. Unresolved debates around accountability and the necessity of patient disclosure and consent for AI use highlighted areas for further guidance.

When discussing pressing ethical concerns of AI in health care use, participants ideated on what constitutes an ethical AI tool. AI tools that are fit-for-purpose and are specific to the future clinical environment where they will be implemented topped the priority list of ethical AI attributes. Experts also raised the concern that AI development and validation are primarily occurring in well-resourced academic medical centers, such as the TMC. These centers already possess the necessary infrastructure, data access, and vendor partnerships to rigorously test these tools. However, implementing AI systems developed in academic medical centers in rural or underresourced settings, where evaluation and validation needs, populations, and systems differ, raises equity concerns. This highlights a growing need for AI models that are specifically designed and validated for use in underresourced areas. Experts also brought attention to the need for continuous monitoring to detect potential data drift. Other participants suggested that AI algorithms should disclose their “list of ingredients” to remain transparent about validation and testing processes. Issues of data privacy were also raised, as well as a need to evaluate algorithms in multiple different “buckets,” including fairness, transparency, and mitigation, among others, and score them before integration. Stakeholders like clinicians and patients should also be involved in the development process to identify the tools that would most benefit them, according to participants.

Focus group participants, and AI developers specifically, agreed that barriers to data access are major blockers to innovation. They highlighted that it is not the amount of data available that is the issue but the administrative hurdles that prevent developers from accessing and using existing data, with 1 developer noting that:

We're drowning in data, we're not taking any advantage of it. We had an amazing amount of information in the different systems…we're just not able to look at the data…all this treasure is sitting there and we're unable to mine it.

Developer, focus group 3

New systems and improved governance that allow for cross-institutional data sharing and support in AI development and validation were cited as potential solutions. Greater collaboration across the TMC was also suggested, as well as appointing a proverbial “chief data evangelist” to manage all the TMC’s data. One administrator expressed that existing governance models were insufficient to address the needs posed by AI, stating that:

One of the challenges that we have is we're not adding new governance for AI. We're trying to make our existing governance AI smart, right? So that's a big challenge for us.

Hospital Administrator, focus group 1

Another central concern raised by experts was the auditability of AI systems, particularly as they become integrated into routine clinical practice. Participants emphasized the importance of continuous auditing to ensure these tools remain accurate and aligned with their intended purposes over time. Several raised critical questions: “How do we know that the system continues to evolve appropriately? How can we enforce auditability once a model becomes ubiquitous in clinical workflows?” A specific concern emerged around the long-term use of AI tools, namely that as practitioners become reliant on these systems, they may lose the ability to critically evaluate or recall pre-AI methods. This reliance could result in fewer individuals capable of conducting unbiased audits. Experts underscored the need for independent auditors who are not overly influenced by the tools themselves and will retain objectivity and the necessary critical distance to ensure AI tools remain safe, effective, and ethical over time.

Some development experts also pointed out the challenge of thinking about ethics during the development process, with 1 developer questioning:

I mean, at some point…you understand there's going to be ethical issues, but you are not ready yet. And it's an interesting point to think when do we become ready? At what point do we think, OK, we can see how this can be used. We can see how this can do good.

Developer, focus group 1

Many also noted that ethics can be out of scope during AI development and that fundamental algorithms can be too abstract and disconnected from applications for conversations around ethics to occur. Another developer expressed that:

We don't expect every developer to be a security expert. We have people who say I'm just going to focus on security and vulnerability. We will need people who think just about ethics.

Developer, focus group 1

Given that individuals have specific roles within AI development based on their expertise, it was agreed that there should be a designated bioethicist on development teams who is responsible for thinking about and handling the ethics of the tool. This would ensure that ethical concerns are kept in mind as early as possible within the AI lifecycle and are not forgotten or left as an afterthought.

Participant discussion also focused on concerns about balancing economic pressures with ethics and patient care. As the health care field is already very costly, experts worry that integrating additional AI systems will burden the system with additional costs and force providers to see more patients in order to recoup them. One developer described this series of events, stating that when you:

Follow this loop, you call it a vicious cycle...then almost a lot of the things that we talked about validating data from AI, double checking, all of this stuff, it has the potential to fall to the side.

Developer, focus group 1

This “vicious cycle,” where AI is integrated to make health care more efficient but then demands that more patients be seen to cover the costs of the technology, which in turn requires even more efficiency, is a major danger of implementing AI tools. Experts also worry that in this scenario, rigorous validation and testing of models will not be thoroughly completed before they are used to attempt to improve efficiency. Participants emphasized that there will be significant tension between the business-industry aspects of running a health care system and the ethical and evidence-based reasoning and decision-making that guide doing what is right.

### Unresolved Questions

#### Shared Accountability vs Provider Responsibility

One issue left largely unresolved among participants was the idea of accountability. Some participants advocated for a shared-accountability model that distributes responsibility across developers, health care systems, providers, and administrators, rather than physicians alone. Although the provider does have final say in clinical decision-making, experts argued that the use of a shared accountability model and continuous monitoring would best ensure the safe and ethical use of AI as a tool in health care. One provider specifically addressed the following when arguing for a shared accountability model:

Health systems can say, oh, we're just going to implement this model and we're going to forget about it. Well, if the model breaks six months later, and your clinicians were now prescribing, I don't know, something wrong, and you did not monitor it as a healthcare organization, well, that's also the healthcare organization's accountability.

Provider, focus group 3

On the other hand, some argued that providers should hold a larger share of the responsibility since AI is still only a support tool used in the decision-making process. Some also noted that a shared-accountability model may leave patients wondering who is responsible for their care, while holding the provider accountable would align with the standard doctor-patient relationship. Moreover, most other participants suggested that as long as providers are the primary decision makers, they should be held responsible for any mistakes that occur under their care. One bioethicist noted that “If the provider is not responsible, then the AI is responsible, which gets into a lot of hairy questions I don't think are really on the table” (Bioethicist, focus group 3), highlighting the challenges and dilemmas that could arise in a nonprovider-accountable system.

Other experts argued for the dangers of excluding providers from responsibility, expressing that providers are less likely to catch mistakes if they are not the ones being held accountable. However, 1 clinician emphasized that focusing on the idea of blame itself is not enough and can actually be unproductive. The focus should not be on identifying the exact error, which can be logistically taxing, but instead on creating systems that enable improvement, prevention, and oversight.

#### Patient Notification of AI Use

Interviewees contested whether and how to disclose AI use in patient care. Preferences for disclosure varied by professional background. Patient advocates and ethicists were often aligned on the principle that patients should be notified of AI use at the point of care, especially when used to make diagnoses, treatment plans, or to communicate through patient portals.

One patient advocate stated:

I think that there should be informed consent on how maybe this [AI] was decided and keeping the patient in check as far as being very clear and concise with how we make decisions for their care and in plain language.

Patient Advocate, focus group 3

Physician participants, however, raised pragmatic concerns about required disclosure, noting that it could overwhelm patients and increase administrative burden. One bioinformatician agreed and also raised concerns about the dangers of requiring disclosure stating:

So, if you're a surgeon, do you need to ask for permission regarding specific suture types? If the patient says no, I want you to use a different suture type, is that what you should be doing, even though you don't think it's the right thing? You know, if you're on, call in the middle of the night and God forbid you look something up. Is that something you should disclose or is it a greater risk to the patient when you didn't look something up and should have?

Bioinformatician, focus group 3

Experts also noted that implementing opt-out systems, where patients could choose to decline AI use, could impede the delivery of standard care and create barriers to accessing care. One physician highlighted that:

Some of these models, I can't, I couldn't think of a way to turn it off as the end users, so we have to be thoughtful about what are the downstream impacts. If they're [a] conscientious objector, does that mean that they can't get care from me? And does that put potential ethical risks for the patient?

Provider, focus group 3

In response, some participants proposed a model similar to the “Gale Model,” where patients are informed about AI use but are not given opt-out options, creating a balance between practicality and autonomy. Others argued that when using AI as a support tool, such as through risk calculators, disclosure is not necessary because the use is not directly patient-facing and instead only assists the physician.

If notification of AI use in care does become the standard—which will occur at the TMC due to recently passed Texas AI legislation—participants noted that communication must be clear and concise to avoid confusion or additional stress. One ethicist emphasized that notification should extend beyond AI disclosure to include potential bias and influence a given AI output may have. Many providers, however, expressed concerns about the practicality of explaining AI outputs and tool use. One provider noted that they anticipated spending a vast majority of their time with patients explaining AI tools, which could take away from the patient care they are able to give. This additional layer to the shared decision-making process risks overwhelming both parties. Therefore, the real challenge lies in determining a balance between providing enough information to support patient and family autonomy in decision making without overwhelming them or placing unnecessary burdens on providers. When it comes to consent for AI, experts highlighted that substantial engagement of stakeholders from all groups should go beyond superficial agreement to terms. One expert noted that just because consent is given does not mean the system is ethically sound, and sometimes patients only consent due to factors such as convenience, not because they truly should. Experts mentioned that as complex AI technologies become more integrated, understanding consent forms and their intent is crucial not only for patients but also for health care providers initiating care.

### Educational Recommendations

When it comes to educating future medical professionals, physician experts stated that it was important to acknowledge that students and residents, regardless of whether they are told to, are using OpenEvidence and other AI platforms. Therefore, it is necessary to figure out a way to incorporate AI use into training so that more experienced physicians can give guidance on how to best use the platform. One suggestion was to integrate clinical decision-making platforms into rounds so that everyone can participate, examine outputs critically, and provide feedback. One physician discussed how she used OpenEvidence in medical education, stating:

One of the things that actually I can provide feedback on is I'll say, what's a question that you have from hearing this patient presentation, you know, put it into open evidence and see what it says. And then based on the response and based on sort of me having like a frame of reference within the practice of medicine, I can actually provide suggestions on how the input maybe should have been framed differently to kind of get at the answer that they're looking for.

Provider, focus group 2

This clinician-scientist also uses OpenEvidence and Copilot to share select papers that align with their daily teaching points and to compose summary emails that attach relevant medical papers and discuss the day’s major lessons. The platform can also be used to write medical examination questions based on clinical vignettes and develop interactive activities based on sample cases that the AI platform can create. Experts also emphasized that educators should focus less on top-down training, which separately delivers training to students, residents, physicians, and others, and rather more on an integrated approach. While generational differences in digital literacy exist, these experts argue that AI training should not be divided by generation and instead be done multigenerationally with exchange and mutual learning between younger students and experienced clinicians, as each group can learn from the other.

Physicians and researchers agreed that clinical experience and judgment are the default, while AI remains supplemental. However, many were concerned that there would come a point where users, and specifically medical trainees, could give AI a degree of trust that is not necessarily warranted, thereby leveraging AI as a replacement for clinical decision-making. One physician described this concern:

We're able to validate a lot of the information that AI is producing...because we have a frame of reference which is experience or you know how we were taught. A first-year medical student who does not have that frame of reference cannot internally validate some of these things. I could potentially see where they could rely on AI to quote unquote do the thinking for them without having the ability to validate the data or validate the output. And this becomes very concerning because if that becomes the norm, then whatever the AI says is correct, because there's no other alternative to think about, right?

Provider, focus group 2

Experts highlighted that these students are still learning and need to grapple with medical concepts themselves, but concerns were raised that students will use AI as a crutch and will not be able to ever discern when AI is wrong. Therefore, education that focuses on developing and testing student’s own reference frames and teaches how to discern where AI recommendations lack validity will be especially important.

Many experts also highlighted that education on AI platforms should focus on transparency and help students understand how the model was developed and how it works. This way, students can be more skeptical and vigilant when applying AI insights to care decisions. Moreover, participants argued that leveraging AI to provide simulations, practice, and case scenarios will become increasingly valuable as physicians face growing pressures from busier practices and other obligations. For example, students can use an AI model trained on senior physician–verified rubrics for history and physical evaluations. Experts noted that these AI models could be particularly helpful as they can provide a form of mentorship that is typically harder to come by, with 1 physician stating:

I think it can serve a really strong role to promote education. Especially when one of our biggest limiting factors in education is the mentorship, the sort of senior person reviewing everything, it's not quite there and there are some portions of that that you know, we can train AI to help with.

Provider, focus group 2

Given the rapid and relatively novel integration of AI into clinical workflows, educating providers, especially those who may lack familiarity with these technologies or feel intimidated by them, is essential to ensuring equitable and responsible adoption. This includes thoughtfully designing onboarding processes and carefully framing communication to support user acclimation. Experts emphasized the importance of training that helps physicians understand how the AI model works so they can effectively respond in rare or unexpected situations. Requiring training or certification for clinical AI use could address this educational gap and ensure a baseline level of knowledge on prompt construction, interpreting AI outputs, and understanding model reasoning. One developer addressed this topic and shared that:

So, in the car industry, for example, Tesla Auto Drive, they have a car inspection industry. They also have a driver’s license industry that ensures the people who use this car get training, actually pass a certain test, so I kind of want to throw a question here to the team. Do we need a regulatory [organization] like FDA to regulate AI models? Or do we also need some certain agent as regulatory for potential users who interact with a model. Do we need that sort of maybe not like a driver’s license? Do we [need] like at least a certificate or something?

Developer, focus group 2

Learning how to unbiasedly prompt generative AI tools can also help address known behaviors in which outputs simply reflect the proclivities and dispositions of the user. Participants also discussed using an integrated approach to health professional training in AI. While generational differences in digital literacy exist, AI training should account for multigenerational learning styles and exchanges.

### Participant Takeaways

When considering the future impact of AI in medicine, participants agreed that patient care should be the priority above all else. As 1 participant indicated, “Remember that medicine has a disease problem, not an AI problem” (Developer, focus group 1), echoing the view of AI as a support tool, not a replacement, of care that should strive to benefit the patient while upholding human dignity. Participants stressed that, similar to many other industries, AI development often has a profit motive that can supersede other stakeholder values. Therefore, focus on ethics must be continuous across all stages of the development and implementation lifecycle. Participants also highlighted that machines are not the ones with ethics; it is the people responsible for their design, implementation, and governance who bear ethical responsibility.

## Discussion

### Principal Findings and Comparison With Prior Work

This study explored how a diverse group of stakeholders within the TMC perceive and experience the practical and ethical challenges of AI use in clinical care. Across focus groups, participants highlighted AI’s potential to extend clinical expertise and support patient care beyond the clinic while simultaneously identifying persistent concerns regarding accountability, auditability, and equity. Key findings also included the tension between innovation and the need for AI-specific governance structures that ensure responsible and ethical deployment, as well as worries of a vicious cycle in which AI use in patient care to increase efficiency ends up demanding more efficiency in turn. Together, these results highlight how real-world AI stakeholders are viewing and experiencing AI in direct patient care roles.

The main aim of this study was to corroborate existing evidence and research on health AI from the perspectives of stakeholders working in a large, regional academic medical center in Texas, where integration of AI in clinical research and workflows is rapidly growing. While the study is grounded in TMC, its contributions extend beyond this setting. The goal of this study was to report on perceptions and direct experiences among key stakeholders with significant expertise or exposure to AI tools. Building on prior conceptual and policy-driven work identifying key ethical and translational considerations for AI in health care [33], this study draws on firsthand perspectives of a diverse range of stakeholders, including providers, developers, administrators, and patient advocates, to identify real-world ethical bottlenecks in AI deployment. Discussions among these stakeholders revealed unique themes and ethical concerns emerging during active AI development and integration. The themes identified are likely to emerge in other large academic settings facing similar pressures and innovation, even if the geographic and regulatory context differs. Therefore, this study contributes empirically grounded ideas that can inform larger efforts to set standards for AI in academic health care settings. Furthermore, this study’s findings have broader relevance to other states that are actively developing or implementing AI regulations in health care. Specifically, states such as California, with similar policies to Texas, as well as states such as Missouri or Ohio, which have not yet developed their own AI regulations, can use these findings to anticipate challenges arising with AI use in patient care under these regulatory settings and to understand what implementing AI legislation actually looks like in terms of effects on patients and health care workers.

With regards to areas for AI development, our findings indicate several priority directions for AI innovation that align with and extend existing work. One area of participant emphasis was the value of tools that focus on encapsulating expertise in areas where it would otherwise be scarce. While prior work has supported AI’s use and potential in improving clinical efficiency and supporting diagnosis [34], this study highlights the underexplored need for AI systems that translate limited expertise across institutional boundaries, specifically to underresourced contexts. Similarly, participants described the use of AI tools that help mediate cross-specialty disagreements, a theme that has not received much attention in the literature but reflects the potential of AI to enhance multidisciplinary decision-making.

Furthermore, consistent with previous work that has demonstrated physicians’ dissatisfaction with the current state of EMRs and information limitations [35], participants suggested integrating LLMs into EMRs to synthesize patient histories, produce and highlight trends, and increase explainability of patient health data. These perspectives reinforce existing work calling for AI chart summarizations but also add new insight into how such tools are greatly desired by physicians and could significantly impact provider experiences and workflows. Other suggestions to develop AI tools that reduce administrative burdens and improve workflows echo prior work identifying clinical inefficiency and clerical tasks as key areas for AI adoption [36]. Stakeholders also saw use for AI tools that provide clinical interactions to patients outside of the clinic. Such tools would help patients better frame questions, interpret test results, and participate more actively in follow-up care [37]. However, concerns were also voiced regarding AI’s risk in patient-facing roles, especially given that models can generalize, hallucinate, or misrepresent uncertainty. This tension reflects broader concerns in the literature about the usability, transparency, and trustworthiness of patients’ use of generative AI models [36]. In short, because of potential patient safety risks posed by models not specifically designed for health care, there is a need for focused AI tools that are validated and designed with patients in mind to minimize unintended consequences.

While prior work has investigated and defined alarm fatigue in health care [38], it has not yet described real-world cases of alarm fatigue due to AI systems. This study also highlights these realities, and our sepsis detection example demonstrates how the problem can be caused by lack of contextual validation. The literature supports the need for validating AI models in the context in which they will eventually be deployed [39], yet we demonstrate that despite these recommendations, validation is occurring primarily in well-resourced hospitals, which creates systemic inequities and a potential digital divide. This finding underscores the need for contextual validation mandates that require models to be designed and tested specifically for underresourced and safety-net settings rather than adopting elite-center models.

One of the most prominent tensions we encountered was over the question of who should be held accountable for AI-related errors, a topic that is widely discussed in the literature yet not accounted for in recent Texas Responsible Artificial Intelligence Governance Act legislation [40]. While some participants preferred shared accountability models that included developers, institutions, and clinicians, others believed that clinicians should retain full responsibility for all aspects of their care. This mirrors disagreements discussed in previous work [41]. As there is no clear consensus on this issue, further research is needed in order to determine legal liability and best practices for implementing AI into clinical practice.

Another unresolved question in the literature [42], which remained unresolved among participants, is the issue of AI disclosure to patients. Current research shows that a majority of patients support AI disclosure [43], although preferences vary based on specific demographic factors [44]. No such research exists on physician perspectives. In our discussion, where clinician perspectives were included, providers were substantially less supportive of AI notification and instead raised many concerns regarding the practical implications of obtaining patient consent while they still retain decision-making authority and remain accountable for outcomes. Providers also worried that notification requirements would add documentation burdens and consume significant amounts of time with patients, thereby hindering overall patient care. These concerns are especially important to address in Texas, where recent legislation mandates disclosure of AI use in clinical care. In this context, one novel framework that emerged from our discussion was a proximity-based scale of notification, where the level of disclosure corresponds to how directly AI influences the care decision or patient communication. In this framework, a physician’s use of a risk calculator or brief consult to OpenEvidence would not warrant disclosure, whereas using AI to generate portal messages or visit notes would. This discussion adds nuance beyond the binary “disclose or not” debates addressed in prior literature.

Furthermore, many findings demonstrate that the concerns raised by stakeholders within the TMC reflect ethical challenges widely discussed in the broader health AI literature. Yet, this work extends these frameworks by grounding them in the daily realities of clinical practice, development, patient experience, and state policy pressures. To situate some of the emerging themes within a structured conceptual framework, we used the FUTURE-AI checklist of principles intended to guide safe and ethical medical AI systems [45]. While our stakeholder findings support and corroborate many of the framework’s key points, they also nuance them by providing real-life examples of how themes play out in direct patient care settings.

First, the framework’s emphasis on fairness was echoed by participant concerns regarding limited data access and disparities between validation in well-resourced and underfunded institutions. Examples of inequitable model performance in underresourced settings reiterate the importance of Universality, the second component of the framework. These experiences provide evidence that issues of fairness and universality are occurring in practice; however, our findings also nuance the framework by demonstrating that inequitable AI performance is not solely caused by algorithmic bias but also by local infrastructure and resource constraints that shape model validation and maintenance.

The stated importance of traceability aligns with findings relating to participant concerns about auditability. Participants highlighted a level of ambiguity regarding who is responsible for continuously monitoring AI systems once embedded in health care settings. This uncertainty demonstrates that it is not just traceability and auditability that are important, but also the governance structures that place explicit responsibility for ongoing evaluation of AI tools. Concerns about physician workflow, documentation burden, and an increased load due to AI tools highlight the importance of usability. While AI is often thought of as a way to improve efficiency, participants specifically noted how AI disclosure to patients and use of tools they are unfamiliar with may significantly increase, rather than decrease, their workload and limit the amount of time they can actually spend on patient care. Additionally, experts noted that integration of AI tools risks creating a “vicious cycle,” in which AI tools increase efficiency while simultaneously raising costs that lead to an even greater need for efficiency and thus less time spent per patient. These concerns demonstrate the potential of AI tools to interfere with health care workers’ ability to accomplish patient care goals, highlighting the importance of tools that are easy to use and actually reduce—not add to—clinician’s workload. Therefore, these findings demonstrate that usability should be evaluated not only in terms of performance and efficiency but also in relation to overall clinician workflow and quality of patient care.

Finally, the explainability component of the framework is validated by debates around provider accountability for AI tools. Given that most participants, though not all, believed that physicians should take full responsibility for decisions made using AI, it becomes of the utmost importance that physicians can understand how systems reach their conclusions in order to fully trust and be willing to take accountability for them. Furthermore, physician comments on the need to build an individual trust and doing personal testing of an AI tool before implementing it into care demonstrates that explainability alone may not be sufficient for a provider to justify trust in a tool. Instead, our findings extend this principle and suggest that personal validation, in addition to explainability, should be expected for providers to fully assume responsibility for a tool in clinical practice.

### Recommendations

This study contributes to the growing body of literature on health AI and highlights expert perspectives on key aspects of integrating AI into the health care system. While prior research has explored ethical frameworks and governance strategies for AI in medicine [27], clear guidelines and recommendations are still lacking, and many barriers remain [46]. Additionally, many concerns have been raised regarding potential problems with health AI tools, including bias [47], transparency [32], and issues in clinical implementation [48], among others. Our findings build on this previous literature by grounding areas of priority and concern in the direct perspectives of developers, administrators, and end users within a large academic medical center and by providing specific recommendations for addressing these issues.

Many participants highlighted the critical equity concern of contextualization and validation of AI models. Discussion focused on how AI models trained and validated in well-resourced settings, as most are today [49], may underperform in areas with fewer resources, exacerbating health disparities [50]. To address this concern, we recommend contextual validation mandates that require the validation of models in a variety of clinical settings and the use of a participatory design with underserved institutions during model development prior to deployment. Participant concerns over a lack of AI-specific governance capable of addressing growing needs highlight existing evidence that new governance bodies specifically designed to address AI concerns are needed [51]. Especially in large academic centers with extensive data, this should include a centralized chief data officer or cross-institutional governing boards that allow for easier data sharing and access. These executives should be responsible for overseeing the development and validation processes to ensure that models are rigorously evaluated before they are used and can affect patient outcomes. Similarly, experts also discussed the need for auditability to ensure systems evolve correctly over time. They also questioned how auditability is enforced once an AI tool is embedded into practice, especially as practitioners become accustomed to the tool. One plausible solution is the creation of independent auditor roles that can perform continuous performance reviews and drift detection. This recommendation parallels other third-party safety boards in many other fields [52].

Our findings also underscore the idea that ethics need to be considered at all stages of the AI lifecycle [16]. This includes the development phase. However, many developers believed that this responsibility should not fall solely on them and that it is an unfair expectation to require coders to be ethical experts. Instead, bioethicists should be embedded throughout the AI development process to provide ethical support and ensure that concerns are raised early in the lifecycle. These bioethicists should also contribute to maintaining ethical standards beyond development and work to ensure that proper validation of the models before implementation. This would provide an additional safeguard on top of recommended AI-specific governance, which would also oversee these activities to minimize risk to patients once the tools are deployed. Stakeholders emphasized the need for clear thresholds for action, or prespecified cutoffs at which AI output or risk scores trigger a specific clinician review or decision. Codevelopment of these threshold guidelines between clinicians, ethicists, and administrators will be essential to ensuring safety and consistency.

Additionally, participants underscored that professional education is an essential part of ensuring ethical AI at the point of care. Previous literature has highlighted the need for training on how to practically use the tools and assess the outputs and ethical implications [53], but in these focus groups, experts highlighted the importance of AI literacy specifically among medical trainees and new medical professionals. As these populations do not possess as much of a frame of reference as more established physicians, they are more susceptible to using AI as a crutch or becoming dependent on it for clinical decision making. Therefore, educational initiatives should focus on having students recognize where AI output may be incorrect or lacking and using established mentors who can demonstrate how they use their own frame of reference to validate AI outputs. For these established providers, education will still be necessary. We recommend developing training on prompt construction as well as required courses on specific AI tools that users must complete in order to use the tool in patient care. These courses would educate physicians on how the tools work to increase transparency and provide the competence needed to use them safely and successfully in clinical practice. Another key point was the need to provide protected time for physicians to individually build trust and experiment with any tools they are going to use. This will help them better understand how the AI tool works, where its output may diverge from the physician’s judgment, and any factors the clinician needs to monitor when using it in practice, thus improving the clinician’s ability to use it safely and effectively in actual patient care.

### Limitations

The 3 focus groups concentrated primarily on issues involving AI development, use, and integration, which restricted the nature and scope of discussions as well as participant responses. Furthermore, the study was conducted in a single, well-resourced academic medical center in Texas. As such, some findings may not be generalizable to rural, nonacademic, low-resourced, or non-Texas settings. Because AI validation and governance challenges, among others, differ substantially between these environments, future work should examine how the ethical and logistical questions identified in this study materialize in other contexts. Resource limitations and time constraints of prospective participants also restricted the number of focus groups that could be conducted. Therefore, the perspectives of stakeholders are not exhaustive nor representative of the totality of professionals working in the TMC. All focus groups were conducted online via Zoom, which may have constrained conversation and influenced group dynamics differently than in-person sessions. Similarly, summer scheduling may have limited participant availability and influenced which perspectives were represented. Additionally, clinicians and developers were more heavily represented in the focus groups, potentially shaping which ethical concerns emerged from discussions. As a result, ethical issues related to patient and administrative groups may be more underrepresented. However, given the study’s exploratory aim to examine how ethical AI issues are emerging in daily clinical and development contexts, the representation achieved was sufficient to surface the key ethical tensions and issues faced by health care professionals today. While this study provides important insights into direct stakeholder experiences within a regional academic medical center, it is not designed to be comprehensive or reach full saturation of perspectives. Instead, it offers an exploratory window into the emerging ethical and practical considerations that matter most to individuals working within the largest medical center in Texas. Further research that includes a wider range of settings and larger stakeholder participation will be necessary to build on the ideas identified in this study.

### Conclusion and Future Directions

This study highlights the current state of AI development and integration in real-world practice within the largest medical center in the country while revealing a number of ethical and practical tensions emerging at the point of care. These findings underscore that AI ethics is not about building an ethical AI machine but about ensuring the ethical development, implementation, and oversight of these tools. Participants’ description of the day-to-day realities of using and developing AI extend the current literature by demonstrating how constraints such as access to data, documentation burden, clinician education shape the true landscape of AI implementation. Furthermore, they demonstrate the importance of independent auditing, AI-specific governance, and embedded ethicists for ensuring current implementation challenges are addressed. This study also demonstrates a need for future work to understand how governance and validation can be standardized across institutions with differing resource limitations. Additionally, quantitative research on the opinions of physicians, patients, and administrators regarding issues of disclosure and accountability would allow for more determinative guidance on the best ways to resolve these debates. Finally, this study highlights critical gaps in workplace preparedness for new health AI systems. Future research could address these by developing empirically grounded curricula for medical trainees and clinicians regarding the use of AI systems, as well as for developers on the ethical development of such systems.

## Data Availability

The datasets generated or analyzed during this study are not publicly available due to confidentiality but are available from the corresponding author on reasonable request.

## Appendix

Multimedia Appendix 1 List of questions asked during our first focus group regarding development and potential use cases for AI in health care.

Multimedia Appendix 2 List of questions asked during our second focus group regarding current real-world AI in healthcare use cases and the problems arriving in their use.

Multimedia Appendix 3 List of questions asked during our third focus group regarding the ethical challenges arising with AI in healthcare implementation.

Multimedia Appendix 4 Additional raw participant data for each results subsection not included in the main text.

## Abbreviations

AI: artificial intelligence
EHR: electronic health record
LLM: large language model
ML: machine learning
TMC: Texas Medical Center
TRAIGA: Texas Responsible Artificial Intelligence Governance Act
