# Supplementary material for: Exploring the Ethical and Practical Considerations of Artificial Intelligence in Real-World Health Care Settings: Stakeholder Focus Group Study
Source: JMIR AI. 2026 Apr 2;5:e85163. doi: 10.2196/85163 (PMC13087557; doi:10.2196/85163)
Supplement: Multimedia Appendix 1 [file ai_v5i1e85163_app1.pdf]

Focus Group 1 Questions

1. What would you say are the most pressing healthcare challenges within the TMC where AI development has been proposed as a potential solution?
2. In what aspects of healthcare delivery and research at your institution do you see the greatest need for AI-driven solutions (e.g., diagnostics, treatment planning, patient management)?
3. What specific AI/ML projects are currently being researched or developed at your institution in response to these problems?
4. What was the motivation for developing these AI tools, did the tool meet your expectations once finished? What were the challenges in this process?
5. Drawing on your past experiences, what do you consider to be essential elements for an effective and ethically responsible AI tool in healthcare? This can be for tools already developed or tools you envision for the future.
6. How were these ethical issues or discussions addressed when developing these AI tools?
7. Who is currently involved in identifying the problems to be addressed by AI systems in healthcare? To what extent are patients, caregivers, and other stakeholders involved in identifying the problems to be addressed and agendas for AI development?
8. What future trends or emerging needs in healthcare should AI developers be preparing for?
9. What should institutions within the Texas Medical Center focus on to create an environment that promotes, safe, effective, state of the art innovation of AI in healthcare tools?
