# Supplementary material for: Exploring the Ethical and Practical Considerations of Artificial Intelligence in Real-World Health Care Settings: Stakeholder Focus Group Study
Source: JMIR AI. 2026 Apr 2;5:e85163. doi: 10.2196/85163 (PMC13087557; doi:10.2196/85163)
Supplement: Multimedia Appendix 2 [file ai_v5i1e85163_app2.pdf]

Focus 2 Group Questions

1. What types of AI tools are you currently using in your role, and what are their primary functions?
2. How has AI changed or improved clinical decision making, workflows, patient care, or research in your field?
    a. What specific patient outcomes or advancements are you anticipating or hoping for when it comes to AI use in patient care
    b. For those who haven't witnessed these improvements yet, what patient outcomes would you be hoping for
3. What impact have you seen AI have on patient outcomes?
4. What constitutes the ethical use of an AI tool at the point of care?
    a. Accuracy, reliability, performance
    b. How do you assess the reliability or accuracy of AI tools in your healthcare practice?
5. How are healthcare providers balancing AI recommendations with their own clinical judgment?
    a. What are some specific challenges that have come about when using and balancing these AI tools in healthcare? What worries you in the respect of balancing AI recommendations with your own clinical expertise?
6. What do physicians and medical trainees need to know about effectively using these AI tools to improve patient care?
7. What has been the response from patients and their families, if any, to the increased or potential use of AI in healthcare? How have you responded or tried to address those concerns?
8. How do you see AI in healthcare evolving in the coming years?
