# Supplementary material for: Exploring the Ethical and Practical Considerations of Artificial Intelligence in Real-World Health Care Settings: Stakeholder Focus Group Study
Source: JMIR AI. 2026 Apr 2;5:e85163. doi: 10.2196/85163 (PMC13087557; doi:10.2196/85163)
Supplement: Multimedia Appendix 3 [file ai_v5i1e85163_app3.pdf]

Focus Group Questions

1. What are the most pressing ethical concerns when integrating AI into healthcare settings?
2. What are the biggest barriers to successfully integrating AI tools into real-world clinical workflows, and how can these challenges be addressed?
3. How can healthcare institutions ensure proper training for clinicians and staff when implementing AI technologies? What do you believe are the most important training needs?
4. Should patients be notified of AI use in their care and what ethical and practical considerations should guide practitioners in this notification?
5. If patients should be notified, what would patients and caregivers need to know to make informed decisions about the use of AI in their care?
6. What are the most ethical ways to integrate systems that are increasingly accurate but limited in their explainability? (what should we be telling patients)
7. How much, or how little, should practitioners rely on AI in clinical decision making and what considerations factor into this decision?
8. What safeguards should be in place to ensure AI tools do not reinforce biases present in healthcare data?
9. Who should be held accountable when AI systems make incorrect or harmful recommendations?
10. What protocols should be put in place to ensure that AI systems are monitored after their deployment to ensure that they continue to meet ethical and clinical standards? Should this be supported by the hospital? Researchers?
