# Supplementary material for: Exploring the Ethical and Practical Considerations of Artificial Intelligence in Real-World Health Care Settings: Stakeholder Focus Group Study
Source: JMIR AI. 2026 Apr 2;5:e85163. doi: 10.2196/85163 (PMC13087557; doi:10.2196/85163)
Supplement: Multimedia Appendix 4 [file ai_v5i1e85163_app4.pdf]

Table S1: Raw data for AI Applications

"Another project is for complex medication management for older adults. There's a lot of drug interactions, but cardiologists, they may just optimize their overall heart health. But as a geriatrician, they care about [the] patient 's overall health. So it's actually a decision [or] debate between different specialists and the role for AI is to objectively collect the information and present evidence to the care team and then to use [a] team based science approach to support shared decision making." - Developer FG2
"Anywhere where patient care can be improved. Or efficiency can be improved and the regulatory obstacles can be surmounted. They're all candidates for AIs, and why do I say that? It's because right now, you know, we do it as humans, as you know professionals. What we try to do is improve patient care. We try to increase efficiency and we try to steer clear of regulatory obstacles. And that's exactly what an AI should be doing too. From my personal perspective, just giving you some candidates. Predictions of no shows and appointments this is a big deal. So to be able to predict them from demographics. Because it's a huge loss of efficiency when someone doesn't show up. There's all kinds of improvements that can be done on the billing side." - Developer FG1
"The other thing…would be an AI tool that you could integrate into an EMR that kind of gives you a sense of what is this patient's frailty? What is their overall cardiovascular health? I think [that] is amazing. My, like, dreams for AI right now in the EMR are much, much lower because what I find the most frustrating thing about working in the EMR is that there is so much information there, and despite the like at least five to six ways that you're supposed to be able to access every piece of information, you still can't always consistently access it…But those are the kinds of things where if it takes AI for us to be able to do that or to be able to search. Even slightly more complex things like, can you summarize the patients cardiac history regarding, you know, imaging, medications, and lab findings with these different criteria over this period of time…that would change clinical practice and that seems small, honestly. But it really would make a huge difference because it would not, it wouldn't give us kind of the answers to clinical questions, but it would make it much easier for us to access the information that we then need to develop clinical answers." - Provider, FG2
"I work in radiology. And there's, you know, if you look at FDA approvals, the vast majority of approved AIs are in radiology. And we have many, many implementations already in our workflow. There's numerous implementations in image acquisition, imagery construction, and in image interpretation. So just in our department, and you know we're pediatric and we're relatively low adoption compared to most adult practices, we probably have 30-40 applications running right now." - Developer, FG3
"One thing that we need to differentiate is the AI model versus the AI products before [the] ChatGPT era. This non generative technology in the back end they upload their medical record, they get a risk score calculation. They get a score. These are, to me, has kind of low, lower risk, but now? Chat GPT [is an] AI model [that] directly disseminates to patients who do not have the knowledge to assess the results and we've done because my background is also in NLP, natural language. We find that the model tends to generalize more assertively and sometimes non factual. But misleading questions directly to the patient users. So I definitely think there's a lot of benefits, but we need to think about converting from the model to a product that the patient can use." - Developer , FG2

Table S2: Raw Data for Implementation Experiences

"[What] I find helpful right now for clinical practice is OpenEvidence, which I find really helpful because it allows me to really quickly kind of explore the literature surrounding a particular topic and it's more helpful than just kind of going to PubMed and looking things up because it kind of allows for combining different aspects of a patient's clinical presentation that you can't really do using PubMed. So there's a lot of ways that I use AI right now. But in the clinical setting, that's probably the main way that I use it." - Provider FG2
"I think when I use OpenEvidence in patient care, I'm using it when I know that I have kind of a clinical question and sometimes it's a very specific clinical question that brings in kind of multiple variables, like it might be a patient who has lupus and has developed an AKI and now they have you know some other symptom and that all kind of comes together in a way that I want to

make sure I'm not missing anything in that differential. And it's difficult to kind of Google that or put it into PubMed and so I can kind of sometimes treat it [OpenEvidence] as almost a colleague to kind of bounce ideas off of and kind of say, well, what would your differential be for this? And then I can make some adjustments like, OK. But, you know, the sodium is this or the, you know, some lab value is this like how would that change your differential? And I can kind of engage with it. And so it's kind of like a built in non judgmental partner in clinical reasoning, which is nice because before I could always turn over to the you know, colleague who's sharing the room with me and just ask them a question about it. But it's a non judgmental kind of safe way to get evidence based information. And the way that OpenEvidence does it that's really helpful, is it actually includes like the citation exactly where it's getting its data from. And there's not the kind of hallucinations and other issues that have come up with like, ChatGPT in particular, when I was kind of trying to do similar things with ChatGPT." - Provider FG2

"You know, again, it's going to be this shared decision making. And I think part of the issue is how the physician is able to communicate AI based outputs in a patient friendly shared decision making model. I don't know if there are guidelines or structure around that. At least I haven't seen it, but I think that's going to become more and more important as these models continue to make it past phase one to phase two…how do we make sure that our patients understand where our decision making is coming from." - Provider FG2

"What ought to be the cut offs and you mentioned how really there's a small window in which you could work in terms of getting it right or getting it wrong. And I wonder [about the] criteria. Are there opportunities to make decisions really about what those benchmarks ought to be as we undertake this?" - Provider FG2

"Rather than just trusting the AI, it can kind of prompt me to ask other people and kind of draws into question my own experience, which is…more limited than a lot of the people that I work with. So it function as kind of like a temperature check for maybe when I should have some degree of discomfort. And maybe for whatever reason I didn't actually recognize that yet." - Provider FG2

"[The] majority of the AI model's nowadays [are] stopped, ended at phase one. People published [a] paper [and] call it from there. It's extremely challenging to translate the AI model to phase two and then after phase two we need to do long term continuous monitoring for potential data drifts." - Developer FG1

"There's been a few models that have come out, as you know, into clinical practice. The one that's gotten probably the most press is this early sepsis detection, which EPIC rolled out as one of their early cognitive computing models. And they showed the performance characteristics and it was OK or pretty decent. And yet when other institutions adopted this model and tried to recreate it, those performance statistics, it was nowhere close to it. And this really goes to that sort of underlying principle that a lot of people have talked about is. You know, a model is only as good as the data that it's fed into. And so when you use hospitals that may have [a] very limited breadth of patient populations or you know unique patient populations, it may work in those populations. But when you try to spread that model to other places where the diversity may be greater or there's just inherent differences between the patient characteristics or where the data is stored, the model is not going to perform very well and so it it gets into another sort of guiding principle, I think within AI, which is you have to validate it before you actually put it into practice." - Provider FG2

"Everybody hates the sepsis alerts. It's just, you know, you've figured out what you need to click to get past it. I don't even honestly pay attention to it at all anymore, because I, I mean, I don't know, maybe I just have this confidence that I can outdo the AI in my own sepsis detection abilities. But it's just that kind of thing, whether it's AI or whether it's just like an algorithm or using [something] like a QSOFA score or whatever it's doing. I think the sepsis alert in Epic and Harris Health has lost everyone's trust. So I think that's a danger of other AI tools as well." - Provider

---

Table S3: Raw Data for Ethical Challenges and Recommendations

---

"Because the resources are in academic centers or large hospitals and so forth and not in these other places…we might have a relationship with a vendor and we could validate it. That vendor is going to go out to a number of hospitals to try to sell their product and in fact those hospitals may be making decisions with inadequate testing and validation because they don't have the

resources to do that. So guidance on really what needs to be done on those implementations, we think about it as a dissemination implementation problem, that may be helpful." - Provider FG3

"Where is AI and the validation going to be done and it's going to be done you know largely in academic medical centers or where there's resources and infrastructure to do it. Sometimes that can be transmitted, you know, to rural communities and so forth…And it would seem that our thinking about that principle comes all the way back to governance." - Provider FG3

"I personally think that we need to evaluate the algorithms in different buckets. In the development bucket, in the fairness bucket, in the mitigation bucket. And then we have to look for applications and score them based on how fair they are, can they be used in a real environment?...We have to look at all the stages of algorithm development…to see whether accountability stands for the whole system, not only on the algorithm. Right. So over the life cycle of development and deployment and maintenance and things like that" - Developer FG1

"So there is a problem that has been obvious now for, at least 15 years going back even before we talk about the AI, we talk about big data, which is we're drowning in data. We're not taking any advantage of it. We had an amazing amount of information in the different systems…we're just not able to look at the data…all this treasure is sitting there and we're unable to mine it…It's not so much a technology right now…The barrier has been more organizational, legal." - Developer FG1

"I think the most important thing is easy access to data. There is no shortage of development and implementation tools. But the big bottleneck is or the small bottleneck is data. So to provide easy access to data is the single most important thing that the institutions can do." - Developer FG1

"So as we get into, you know, AI being more commonplace, it will be ubiquitous and we'll get very comfortable with it. And when we get to that stage, you know, I'm inclined to ask, how do we know that we're continuing to train the models and the bots and that they continue answering the questions that we posed to it accurately? In technology, one of the things that we do is we continuously audit systems and we monitor systems to make sure that they're performing at the level or the thresholds that we expect and when they dip below, then we intervene. Have we thought about things like that as we, you know, bring AI really to the land of ubiquity, and that's what I would want to pose. How do we know? I think I might liken it to continual medical education. How do we know that the bot is continuing to develop?" - Hospital Administrator FG1

"Now I want to come back to the question of auditability, that [participant] raised here. And you know the way to do an audit, of course, is for a human to look at the predictions of the results of the machine and see if it is correct. In other words, you have a gold standard that is human based. The challenge here is one effect limitation. This is a well known phenomenon that when you have tools deployed in a practice setting, as time goes on, the practitioner becomes used to the tool and forgets how it was done before the tool existed. And so with the passage of time, we're going to find that our auditors are going to become rarer and rarer. And so if we really want to follow an audit methodology. We're going to have to come up with auditors that are not mentally plastic." - Developer FG1

"So if you have people who develop very, very fundamental algorithms. You know, it's just too abstract and disconnected from applications…it's very hard to discuss responsibility and ethics in terms and and trustworthiness in absence of concrete context…I mean, at some point…you understand there's going to be ethical issues, but you are not ready yet. And it's an interesting point to think when do we become ready? At what point do we think, OK, we can see how this can be used. We can see how this can do good." - Developer FG1

"You can't expect every coder to become an expert on ethics. Ok, so the team will have to have the people develop systems…and we have people who think about just the security aspect of the system. And we don't expect every developer to be a security expert. We have people who say I'm just going to focus on security and vulnerability. We will need people who think just about ethics." - Developer FG1

"So I want to double down on what my friend said already, but basically I see the value of the bioethicist in the team to first educate the coders in terms of the ethical [aspects]. And of course, I see that later on we're going to have multiple categories in terms of functional validation, but I want to come back on the issue of education and I think the bioethicist in the team is going to do this initial education." - Developer FG1

“And then the third challenge is that much of our medical industry is for profit…and yes, they are driven by profits, but they still, even under these profits, they have to follow these two guidelines: benefit of the patients and human dignity. And in that sense, AI is not different. It's just yet another tool. It's going to be developed by for-profit corporations, and we have to insist that even when you do..these two other guiding principles [we] will never forget about them.” - Developer FG3

“AI is not free. AI costs money and there's going to be a strong push from a business industry, you know, machine-like process that sometimes medicine can feel like. And so, you know, I've even heard conversations around, well, we are going to deploy ambient AI to take notes for the patients, for the physicians that costs money. And there's not much room in medicine to kind of just absorb these costs. So the way that it's going to be recouped is by seeing more patients, and you follow this loop, you call it a vicious cycle, you know, continue forward, then almost a lot of the things that we talked about validating data from AI, double checking, all of this stuff, it has the potential to fall to the side. And there is a potential and I think when you mentioned this as well that just use the AI to make you more quote on quote efficient and then you get to see more patients and you get to this sort of downward spiral where we are kind of using AI a lot more in our in our practice to quote on quote make things efficient. But are we truly holding, you know, true to our core values of practicing medicine and patient centered and all of these things? So I think there's going to be a lot of tension between sort of the money business side of running a hospital and healthcare system versus sort of the ethical kind of do what's right evidence based sort of side of things and that sort of tension I think is only going to grow.” - Developer FG3

Table S4: Raw Data for Unresolved Issues

“I think this is gonna end up being a need for a shared accountability model…it's a shared responsibility between the developers, the health systems, and their clinicians and even other external stakeholders. And it's going to be the same for AI as well. I know ultimately everybody's going to try to push it to the physicians like [participant] was telling us, but it’s going to end up being more than that because there are several instances where you shouldn't really be using a bad model, or you should be really monitoring the model you are supposed to be using so health systems can say, oh, we're just going to implement this model and we're going to forget about it. Well, if the model breaks six months later, and your clinicians were now prescribing, I don't know, something wrong, and you did not monitor it as a healthcare organization, well, that's also the healthcare organization's accountability. So we're going to have to sort of think about this shared accountability model.” - Provider FG3

“Seems to me the whole argument that's been to place the responsibility on the physician involved or the healthcare provider as who that may be, because in this discussion AI has strictly been a tool. So to question whether I mean the alternative, of course is if the provider is not responsible, then the AI is responsible, which gets into a lot of hairy questions I don't think are really on the table, so it seems to me that the whole argument has been it's the provider that's responsible because the provider is using it as a tool, even if it's the standard of care, because standard of care is just a guideline. It's not intended to be an absolute rule because the physicians retain professional insight or whatever into what they're doing.” - Bioethicist FG3

“There was a book by Dr. Walker where he cited a case where there was a doctor who put in I think pounds instead of kilograms per child patient, and there was a robotic pharmacy that dispensed 24 septra tabs. I believe that was an antibiotic and the nurse was a floating nurse and didn't know the patient and gave it to the child. And the child was basically overdosed on antibiotics and Epic didn't catch it. So when you take the providers out of the loop, I think that's where the problems are going to start.” - Patient Advocate FG3

“Yes, the physician gets to use AI in their practice. But here now, if we start looking, we're questioning a kind of from a different context from saying who's responsible for this treatment, who's you know, who, who at the end of the day is going to be responsible. And if you want [a] shared responsibility model, which you know that whole argument was. It’s been around for quite some time, then I think that it bears on what you tell the patient because patients have a right to know who's responsible for their treatment.” - Bioethicist FG3

“I do think that patients should be notified just like we have to have them sign consents and we also have to ask permission when we're at a provider, if we can either record or take notes or try to introduce ourselves. So when there's, you know, informed decision making between the physician and the patient. I think that there should be informed consent on how maybe this [AI] was

decided and keeping the patient in check as far as being very clear and concise with how we make decisions for their care and in plain language.” - Patient Advocate FG3

“I think it depends on the use case of that but say we're using a generative AI tool to help in creating a differential diagnosis similar to what I would be doing if in the middle of the night I'm going to UpToDate and try to find a differential diagnosis…What is our responsibility if the clinician is still in the loop and it's [AI] helping to support the clinician's decision making and it's not necessarily generating content that the patient is receiving, is that still something that we are required to disclose because…I would argue that that type of AI is not needed to have consent.” - Provider FG3

“So if you're a surgeon, do you need to ask for permission regarding specific suture types? If the patient says no, I want you to use a different suture type, is that what you should be doing, even though you don't think it's the right thing? You know, if you're on, call in the middle of the night and God forbid you look something up. Is that something you should disclose or is it a greater risk to the patient when you didn't look something up and should have? Should you disclose that you didn't look something up for all decisions that you make? Where does this end right? ” - Bioinformatician FG3

“If we're consenting them, isn't there an opportunity for them to opt out? And with what we're just talking about is there a way to opt out of that as a patient? I can hear if we're using general AI to generate my clinic notes, I can just not turn on the transcribing. But, some of these models, I can't, I couldn't think of a way to turn it off as the end users, so we have to be thoughtful about what are the downstream impacts. If they're [a] conscientious objector, does that mean that they can't get care from me? And does that put potential ethical risks for the patient?” - Provider FG3

“So I think it's a little hard to answer this question. When you lump all of AI together. because there's so many different tools that are used in so many different ways and for different purposes, some of them I think of which require sort of explicit disclosure and maybe even consent and others of which don't, because they're just part of, like the mechanics of the you know, decision making process and are taken into consideration along with a lot of other factors.” - Policy Expert FG3

Table S5: Raw Data Educational Recommendations

“So students and residents are using open evidence, right? Regardless of whether we tell them to or whether we're supervising it. And once I realized that I was like, we need to just integrate this into rounds so that everybody can see what the output is. We can examine it critically. And we can provide feedback…one of the things that actually I can provide feedback on is I'll say, what's a question that you have from hearing this patient presentation, you know, put it into open evidence and see what it says. And then based on the response and based on sort of me having like a frame of reference within the practice of medicine, I can actually provide suggestions on how the input maybe should have been framed differently to kind of get at the answer that they're looking for as well.” - Provider FG2

“How do you use AI appropriately to help education and an early trainee without making it a default or a crutch? You know, one of the things that I think [other provider] and I have the benefit of is we're able to validate a lot of the information that AI is producing. We're able to look at it and say, yeah, this is what I was thinking. This is along the right lines. This sounds right. And we can because we have a frame of reference which is experience or you know how we were taught. A first year medical student who does not have that frame of reference cannot internally validate some of these things. I could potentially see where they could rely on AI to quote unquote do the thinking for them without having the ability to validate the data or validate the output. And this becomes very concerning because if that becomes the norm, then whatever the AI says is correct, because there's no other alternative to think about, right?” - Provider FG2

“I think it will be beneficial for [medical students] to understand this current state of how [the] AI model is developed. [That] they are trained based on prior imperfect data sets. They make inferences. The larger language model is just predicting the next token or character given the prior sentence. They don't do. They don't think. They don't do reasoning. It's a kind of false sense of intelligence given to people, but they are actually just statistical models trained, the entire thing on the Internet. I think that would help them be more skeptical and vigilant about when they use the AI.” - Developer FG2

“I think it can serve a really strong role to promote education. Especially when one of our biggest limiting factors in education is the mentorship, the sort of senior person reviewing everything, it's not quite there and there are some portions of that that you know, we can train AI to help with. And when I say train, I want to be very clear. It's not just typing it into Chat GPT and saying giving me feedback, but you give ChatGPT the rubric that the senior clinicians have kind of come up with and these are the things that we would look for and then it uses that rubric and guides it. So I think there are some really strong use cases in the education world.” - Provider FG2

“Transparency and interpretability, so whether the clinicians or health professionals can understand, you know how to use it. So if the data has some extreme cases, for example like outliers or distribution shift, then you know [how] the model works in that kind of unexpected situation.” - Developer FG1

“I think anyone who's using AI could greatly benefit from some baseline level of education on things like prompt development or how to interpret outputs, or how to engage in a back and forth with an AI.” - Provider FG3

“I think more than most of us, and so not only will our next generation of clinicians and providers be much more facile and and familiar and comfortable with all kinds of uses of AI, um, appropriate and inappropriate, maybe for for the healthcare setting, but also patients are are going to have facility um with different AI tools as well, and so it might be that those of us who have been in healthcare for a long time and the institutions that have leaders that have been in healthcare for a long time may have to be um in some way trained by those coming in, uh, who are of the next generation. And so I think it has to be a partnership in training and it can't be a top down approach.” - Policy Expert FG3

“So in the car industry, for example, Tesla Auto Drive, they have a car inspection industry. They also have a drivers license industry that ensures the people who use this car get training actually pass a certain test, so I kind of want to throw a question here to the team. Do we need a regulatory [organization] like FDA to regulate AI models? Or do we also need some certain agent as regulatory for potential users who interact with a model. Do we need that sort of maybe not like a drivers license? Do we like at least a certificate or something?” - Developer FG2

Table S6: Raw data for Participant Takeaways

“So I wrestled with this issue of ethical AIs all the time, because at the end of the day it's a whole calculating machine, it has no ethics. Ethics are a social construct. So it's the people who implement the machine that are really in charge of the ethics. So I don't think we should talk about ethical AI's. We should talk about ethical use, ethical development, ethical implementation.” - Developer FG1

“You could imagine building rules into the technology. OK, you can build rules into the technology to discover bias. You can imagine it is built into the technology, OK, but the responsibility and the ethics belong to the people who develop the technology and use the technology. Not to the technology. We should not confuse the two things. People are supposed to be ethical and responsible. Machines are just machines.” - Developer FG1

““There's a ton of collaboration happening today across the TMC. It's wonderful. That really needs to continue and we need to keep investing in it…So comparing notes with other institutions around the TMC would be very helpful. So I really do continue pushing for that collaboration and identifying opportunities for folks to further compare notes.” - Hospital Administrator FG1
